# Multiomic insights into the MPO-mediated NET formation pathway in alcohol-induced epilepsy risk

**DOI:** 10.1016/j.gendis.2025.101917

**Published:** 2025-11-01

**Authors:** Ningning Zhang, Sirui Chen, Jialing Jiang, Hong Jiang, Qing Wang, Srikrishnan Raju, Jackson G. Schumacher, Jiliang Lu, Yihe Lian, Yuansong Zhang, Yuanhang Xu, Lan Zhang, Yaqing Liu, Junqiang Li, Yiru Zhang, Yuxuan Wang, Yixue Gu, Tiancheng Wang, Xin Tian

**Affiliations:** a The Second Hospital & Clinical Medical School, Lanzhou University, Lanzhou, Gansu 730030, China; b Department of Geriatrics, Laboratory of Research and Translation for Geriatric Diseases, The First Affiliated Hospital of Chongqing Medical University, Chongqing 400016, China; c Department of Epilepsy Center, The First Affiliated Hospital of Chongqing Medical University, Chongqing 400016, China; d CQMU-University of Leicester Joint Institute, Chongqing Medical University, Chongqing 400016, China; e Department of Neurology, Massachusetts General Hospital and Harvard Medical School, Boston, MA 02129, USA; f Department of Dermatology, The First Affiliated Hospital of Nanjing Medical University, Nanjing, Jiangsu 210029, China; g Department of Neurology, The First Affiliated Hospital of Chongqing Medical University, Chongqing Key Laboratory of Major Neurological and Mental Disorders, Chongqing 400016, China; h Key Laboratory of Major Brain Disease and Aging Research (Ministry of Education), Chongqing Medical University, Chongqing 400016, China; i Department of Neurology, Epilepsy Center, The Second Hospital of Lanzhou University, Lanzhou, Gansu 730030, China; j The First Clinical Medical College, Chongqing Medical University, Chongqing 400016, China

**Keywords:** Alcohol consumption, Epilepsy, MPO, Multiomic analysis, NET formation pathway

## Abstract

Epilepsy is a highly prevalent chronic central nervous system disorder that imposes substantial societal and economic burdens. Inconsistent associations of alcohol consumption, identified as a major global health risk factor, with epilepsy risk have been reported. The aim of the present study was to assess the relationship between alcohol use and epilepsy and to identify potential underlying mechanisms, with a particular focus on the role of neutrophil extracellular traps (NETs), using an integrated multiomic approach. We assessed the global risk of alcohol consumption for epilepsy using data from the Global Burden of Disease Study 2021, and we conducted a Mendelian randomization (MR) analysis to evaluate causality. Additionally, we employed machine learning algorithms and protein–protein interaction networks to identify key genes. Our results indicate that alcohol consumption significantly contributes to the risk of epilepsy, as confirmed by MR analysis (odds ratio = 1.30, 95% confidence interval 1.06–1.60; *p* = 0.011). Functional enrichment analysis revealed pathways related to NET formation, whereas machine learning identified key genes such as myeloperoxidase (MPO) and neutrophil elastase. Animal and molecular experiments confirmed that acute alcohol exposure increases the susceptibility to epileptic seizures, whereas the MPO inhibitor 4-aminobenzoic acid hydrazide showed therapeutic potential for alcohol-induced epilepsy. This study provides novel insights into the role of NETs in alcohol-induced epilepsy and highlights potential therapeutic targets, thereby contributing to the development of innovative treatment strategies for epilepsy prevention and management.

## Introduction

Epilepsy is among the most prevalent chronic central nervous system disorders and is characterized by recurrent episodes of behavioral changes and seizures.^1^^,^^2^ Epilepsy affects individuals across all age groups and social classes, imposing a substantial burden on families and society. This burden is attributed to the association of epilepsy with high economic costs, social stigma, and psychiatric comorbidities.^3^^,^^4^ According to the 2016 Global Burden of Disease (GBD) Study,^5^ nearly 50 million people worldwide have idiopathic or secondary active epilepsy. Additionally, approximately 12.6 million epilepsy-related deaths and 13.5 million disability-adjusted life-years (DALYs) were reported. Multiple risk factors influence the onset of epilepsy, and the effects of lifestyle factors, including alcohol consumption, are particularly notable.^6^

Alcohol consumption is a significant risk factor for the global burden of disease and is a prevalent dietary habit in daily life. Alcohol consumption has been shown to increase the risk of various diseases, such as cancer, stroke, and cardiovascular diseases.^7^^,^^8^ Moreover, alcohol-related injuries, including traffic accident injuries and interpersonal violence, are major public health concerns.^9^ Numerous prior studies have revealed an association between alcohol consumption and the risk of epilepsy; however, the results have been inconsistent.^10^, ^11^, ^12^ Overall, several recent Mendelian randomization (MR) studies and existing meta-analyses of case‒control studies have indicated that alcohol consumption has a detrimental effect on epilepsy risk.^13^, ^14^, ^15^, ^16^ Conversely, some cohort studies have reported inconsistent findings, with moderate alcohol consumption either reducing the risk of epilepsy or having no effect on seizure frequency.^10^^,^^17^ Currently, the mechanisms through which alcohol consumption affects epilepsy primarily involve changes in neurotransmitter levels in the brain, metabolic disorders in the brain, direct neuronal toxicity, and genetic influences.^18^^,^^19^ Despite these findings, the effects of alcohol consumption on epilepsy and other potential mechanisms remain underexplored.

Neutrophil extracellular traps (NETs) represent a unique form of the innate immune response in which neutrophils undergo a specialized form of programmed cell death. During this process, neutrophils extrude elaborate reticular structures into the extracellular space. NETs are composed of depolymerized chromatin decorated with an array of highly cytotoxic granule-derived proteins. Key constituents include histone proteins, depolymerized genomic DNA, granulocytes, neutrophil elastase (ELANE), antimicrobial peptides, and myeloperoxidase (MPO).^20^^,^^21^ Abnormal NETs have been implicated in the pathogenesis of various diseases, including immune-related disorders,^20^ cardiovascular diseases,^22^ and tumors.^23^ In recent years, the role of NETs in the nervous system has garnered increasing attention; however, research has focused primarily on meningitis,^24^ ischemic cerebrovascular disease,^25^ and traumatic brain injury.^26^ Under these conditions, NETs exert their effects through multiple pathways, including disruption of the blood‒brain barrier, endoplasmic reticulum stress, neuroinflammation, and neuronal apoptosis.^24^^,^^26^, ^27^, ^28^ To date, the role of NETs in the pathogenesis of epilepsy remains largely unexplored. However, given that excessive alcohol consumption increases neutrophil infiltration and oxidative stress^29^ and that alcohol can directly trigger NET formation,^30^ NETs may play a crucial role in the association between alcohol consumption and epilepsy.

**Figure 1:**
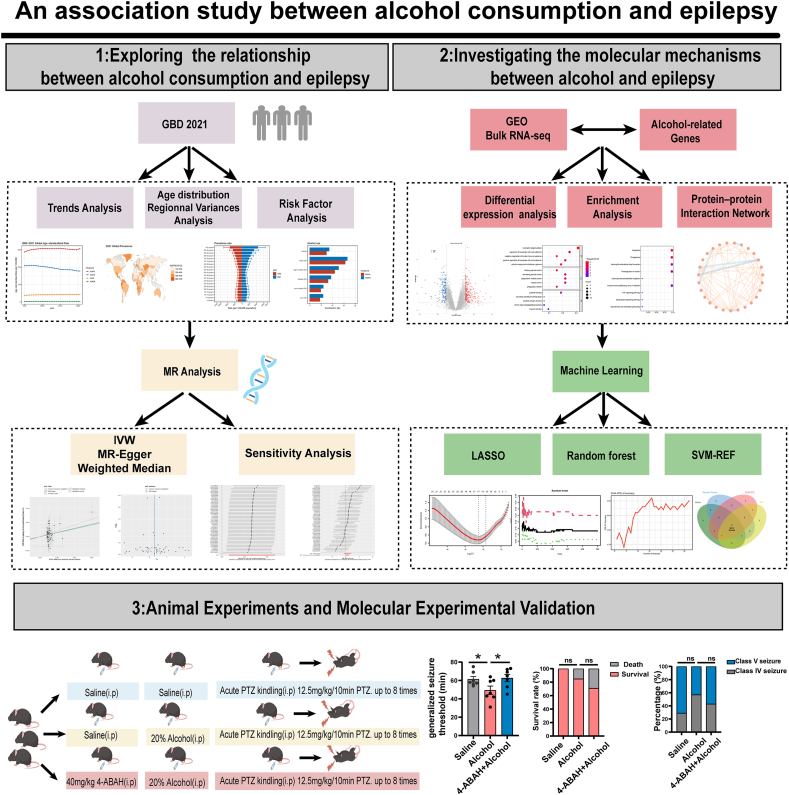
Analytical workflow for investigating the potential risk of epilepsy associated with alcohol consumption.

In GBD studies, the severity of and risk factors for major diseases across countries are quantified in a highly standardized manner, aiming to provide an efficient and timely assessment of clinical outcomes for key health issues.^31^ The Gene Expression Omnibus (GEO) database is an international public repository of gene expression data, featuring abundant, high-quality, and diverse data types.^32^ In the protein–protein interaction (PPI) network, the STRING platform is utilized to screen and identify crucial modules and hub genes.^33^ Machine learning (ML), a subfield of artificial intelligence that integrates statistics and computer science, involves the construction of predictive algorithms by autonomously learning from large gene expression datasets. The methods most frequently employed for identifying key genes include least absolute contraction and selection operator (LASSO), random forest (RF), and support vector machine–recursive feature elimination (SVM-RFE).^34^ Molecular experiments combined with behavioral tests in animals further identified the key functional molecules involved. The aim of this study is to explore the effects of alcohol consumption on epileptic seizures and the underlying mechanisms by integrating these different statistical methods and basic experimental verification and to emphasize the potential role of MPO in this process. An overview of the study is depicted in Fig. 1, which was created using BioGDP.com.^35^

## Materials and methods

### Global risk of alcohol consumption for idiopathic epilepsy

In this study, data from the GBD 2021 database, a highly authoritative resource for analyzing the global health burden (https://www.healthdata.org/gbd), were leveraged to evaluate the risks associated with alcohol consumption and idiopathic epilepsy. Through our analysis, we examined trends in idiopathic epilepsy incidence, prevalence, and DALYs across various regions, sociodemographic index (SDI) quintiles, and groups stratified by sex and age. We also employed risk factor analysis to determine the percentage contribution of alcohol consumption to idiopathic epilepsy DALYs. By utilizing comprehensive and standardized data from the GBD, we aimed to provide critical insights into the global risk factors for idiopathic epilepsy, thereby informing relevant prevention and intervention strategies.

### Mendelian randomization analysis

MR is a method in which single nucleotide polymorphisms (SNPs) are used to robustly assess the causal effects of exposure factors on outcomes, largely overcoming the effects of confounding factors in observational studies, and is widely applied in various clinical causal inferences. To further assess the causal relationship between alcohol consumption and epilepsy, we conducted a two-sample MR analysis. The MR must satisfy three fundamental assumptions^36^: (1) instrumental variables (IVs) are strongly associated with exposures; (2) IVs are not associated with confounders; and (3) IVs influence the outcome only through exposures, with no other pathways.

The summary statistics of IVs for alcohol consumption, smoking, obesity-related indicators, and epilepsy were derived from publicly available genome-wide association study (GWAS) data. Data for epilepsy were sourced from the International League Against Epilepsy consortium cohort, comprising 15,212 epilepsy cases and 29,677 controls. IVs for alcohol consumption and smoking (including alcohol consumption and smoking per day) were extracted from the Genome-wide Association Study and Sequencing Consortium of Alcohol and Nicotine Use.^37^ Obesity-related indicators, such as waist circumference, hip circumference, waist-to-hip ratio, and body mass index (BMI), were extracted from other publicly accessible GWAS databases.^38^^,^^39^ To identify independent SNPs strongly associated with exposure, we performed clumping with a threshold of 1000 kb and a maximum linkage disequilibrium (*r*^2^) of 0.01. We excluded SNPs with palindromic features and those with intermediate allele frequencies. Additionally, the F statistic was employed to gauge the statistical power of the MR analysis.

In our primary analyses, we utilized the inverse-variance weighted (IVW) method as the main approach, complemented by supplementary analyses including weighted median, MR‒Egger, simple mode, and weighted mode. To evaluate heterogeneity, both IVW and MR‒Egger regression analyses were performed, with Q statistics used to measure the extent of heterogeneity. Horizontal pleiotropy was assessed using MR‒Egger intercept analysis. Additionally, the Mendelian randomization pleiotropy residual sum and outlier (MR-PRESSO) global test was utilized to explore total pleiotropy and identify any anomalous SNPs.

### Differential expression analysis

The dataset for analyzing differentially expressed genes (DEGs) was sourced from the GEO database (https://www.ncbi.nlm.nih.gov/geo/). The GSE143272 dataset comprises 91 samples from epilepsy patients and 51 control samples obtained from human blood.^40^ To identify the DEGs between the epilepsy and control samples, we conducted differential expression analysis using the “limma” R package. The criteria for identifying DEGs were a |log_2_Fold Change| > 0.26 and a *p* value < 0.05.

### Alcohol-related genes

Alcohol-related genes were identified using the GeneCards database (https://www.genecards.org/). We selected genes that were both associated with alcohol and differentially expressed in epilepsy, and intersecting genes were earmarked for further analysis.

### Functional enrichment analysis

We conducted an enrichment analysis on the genes common to both the alcohol-related and epilepsy DEG sets to clarify their functional context. We utilized the “clusterProfiler” package in R for Gene Ontology (GO) and Kyoto Encyclopedia of Genes and Genomes (KEGG) pathway analyses. This method enables a comprehensive evaluation of key module eigengenes and their roles in biological pathways, revealing the potential molecular mechanisms through which alcohol consumption affects epilepsy.

### Protein–protein interaction network and importance ranking

We constructed a PPI network using the STRING database (http://string-db.org/) with a confidence score threshold of >0.4. The network was visualized and analyzed using Cytoscape software (version 3.10.1), which allowed us to map the interactions and assess the connectivity of the nodes. To identify important modules within the PPI network, we employed the Molecular Complex Detection plugin in Cytoscape. This algorithm identifies highly interconnected regions within the network, suggesting functional complexes or modules. Additionally, the Cytohubba plugin was utilized to identify hub genes on the basis of network topology metrics such as degree, betweenness centrality, and closeness centrality.

### Machine learning

ML integrates statistics and computer science to develop predictive algorithms that can autonomously learn from large gene expression datasets, thereby identifying key genes. In our study, we applied several prominent ML techniques—LASSO, RF, and SVM-RFE—to identify key alcohol-related genes associated with epilepsy. The genes selected using each method were then intersected to identify a consensus set of alcohol-related hub genes that were consistently highlighted as important across the different algorithms. To rigorously evaluate the predictive performance of the identified consensus alcohol-related hub genes in distinguishing epilepsy patients from controls, we constructed a receiver operating characteristic curve and calculated the corresponding area under the curve. A predictive nomogram was subsequently developed to predict epilepsy risk, and the precision of the model was validated using calibration and decision curve analysis. For validation, we used the GSE16969 and GSE32534 datasets,^41^^,^^42^ both of which utilize the GPL570 platform. These datasets were merged into a single cohort, and batch effects were corrected to avoid bias.

### Basic experimental protocol

#### Experimental animals and ethical statements

Male C57BL/6J mice (6–8 weeks old) were procured from the Laboratory Animal Center at Chongqing Medical University, China. The animals were randomly allocated to experimental groups and maintained under specific pathogen-free conditions with controlled temperature/humidity and a 12-h light/dark cycle, with ad libitum access to food and water. All experimental procedures were approved by the Ethics Committee of Chongqing Medical University and performed in compliance with the Guidelines for the Care and Use of Laboratory Animals issued by the National Health Research Institute.

#### Acute alcohol exposure model

To investigate the effects of acute ethanol exposure on hippocampal protein expression in mice, the animals were randomly allocated into two groups. The ethanol-treated group received an intraperitoneal injection of 20% ethanol solution (4 g per kilogram of body weight),^43^ whereas the control group was administered an equivalent volume of saline. Six hours post-injection, all the mice were euthanized for brain tissue collection.

#### Acute alcohol exposure in a chronic KA-induced epilepsy model

To evaluate the effect of acute alcohol exposure on protein expression in the hippocampal tissue of mice with chronic epilepsy. Under anesthesia, the mice were secured in a stereotaxic apparatus (RWD Life Science Co. Ltd., China). Kainic acid (KA) (0.3 μg/0.5 μL saline; Sigma–Aldrich, 487-79-6) was subsequently delivered over 3 min into the right CA1 hippocampus (coordinates relative to the bregma: ante roposterior, 2.0 mm; mediolateral, 1.5 mm; dorsoventral, 1.5 mm).^44^ Status epilepticus developed within 2 h post-injection and was pharmacologically terminated with diazepam (10 mg/kg, i.p.). Thirty days later, the chronic KA-induced epilepsy model was established. The successfully established mice were subsequently divided into two groups. The ethanol intervention group was intraperitoneally injected with 20% ethanol (4 g per kilogram of body weight), whereas the control group was injected with the same volume of normal saline. After 6 h of intervention, the mice were euthanized, and their brain tissues were collected.

#### Acute alcohol exposure with PTZ-induced seizures

To examine the effects of acute ethanol exposure on hippocampal protein expression and seizure behavior in an acute epilepsy model, the mice were pretreated with either 20% ethanol or an equivalent volume of saline for 6 h prior to convulsive PTZ (54-95-5; Sigma–Aldrich) testing. Seizures were induced through serial intraperitoneal injections of pentylenetetrazol (PTZ, 12.5 mg/kg per administration) at 10-min intervals, continuing until manifestation of generalized convulsions (Racine stages IV-V) or attainment of the maximum cumulative dose (100 mg/kg).^45^ Comprehensive seizure assessment according to the Racine scale,^46^ with specific parameters quantified, including (1) initial latency to generalized seizure onset (stage IV/V), (2) incidence rate of PTZ-triggered generalized seizures, (3) experimental mortality rate, and (4) the proportion of subjects exhibiting high-grade seizure activity (Racine scores IV-V). Upon completion of the behavioral assessments, the mice were euthanized, and their brain tissues were collected.

#### Drug administration

To further investigate the effects of the MPO inhibitor 4-aminobenzoic acid hydrazide (4-ABAH) on seizure behaviors during acute ethanol exposure combined with PTZ-induced epilepsy, a sequential intervention protocol was designed. First, the mice were intraperitoneally administered 4-ABAH (40 mg per kilogram of body weight).^47^ Two hours later, they were injected with 20% ethanol solution (4 g per kilogram of body weight). Following an additional 6-h interval, the acute PTZ-induced seizure model was established as described above, after which behavioral characteristics were recorded. This intervention is anticipated to attenuate the proconvulsant effects of acute ethanol exposure on PTZ-induced epileptic seizures.

#### Protein extraction and Western blot analysis

Hippocampal tissues were isolated from mouse brains and homogenized in RIPA lysis buffer (Beyotime Biotechnology, China) supplemented with PMSF, followed by 30 min of incubation on ice and centrifugation at 16,000× *g* for 15 min. Protein concentrations were quantified using an enhanced BCA assay kit (Beyotime), with denaturation performed in 5 × SDS loading buffer (Beyotime). Proteins were resolved using SDS‒PAGE (Epizyme Biotechnology) and transferred onto 0.45 μm polyvinylidene difluoride membranes (Millipore, Billerica, MA, USA). After 1 h of blocking with 5% skim milk in TBST at room temperature, the membranes were probed with primary antibodies, including those against GAPDH (Proteintech, 81640-5-RR), MPO (ABclonal, A24531), and ELANE (ABclonal, A13015), at 4 °C overnight. This was followed by incubation with HRP-conjugated secondary antibodies. The protein bands were visualized using Western Bright ECL (Advansta, USA) and imaged with a Fusion FX5 system (Vilber Lourmat, France).

### Statistical analysis

All the statistical analyses were conducted using R software (version 4.3.1) and GraphPad Prism (version 8.0.1). Differences in variables between two groups were assessed using an unpaired two-tailed Student's *t-*test and the *χ*^2^ test. Significant differences are indicated as follows: ∗*p* < 0.05, ∗∗∗*p* < 0.001, and ∗∗∗∗*p* < 0.0001, whereas nonsignificant differences are indicated by the abbreviation “ns”.

## Results

### Alcohol consumption may contribute to the risk of epilepsy

**Figure 2:**
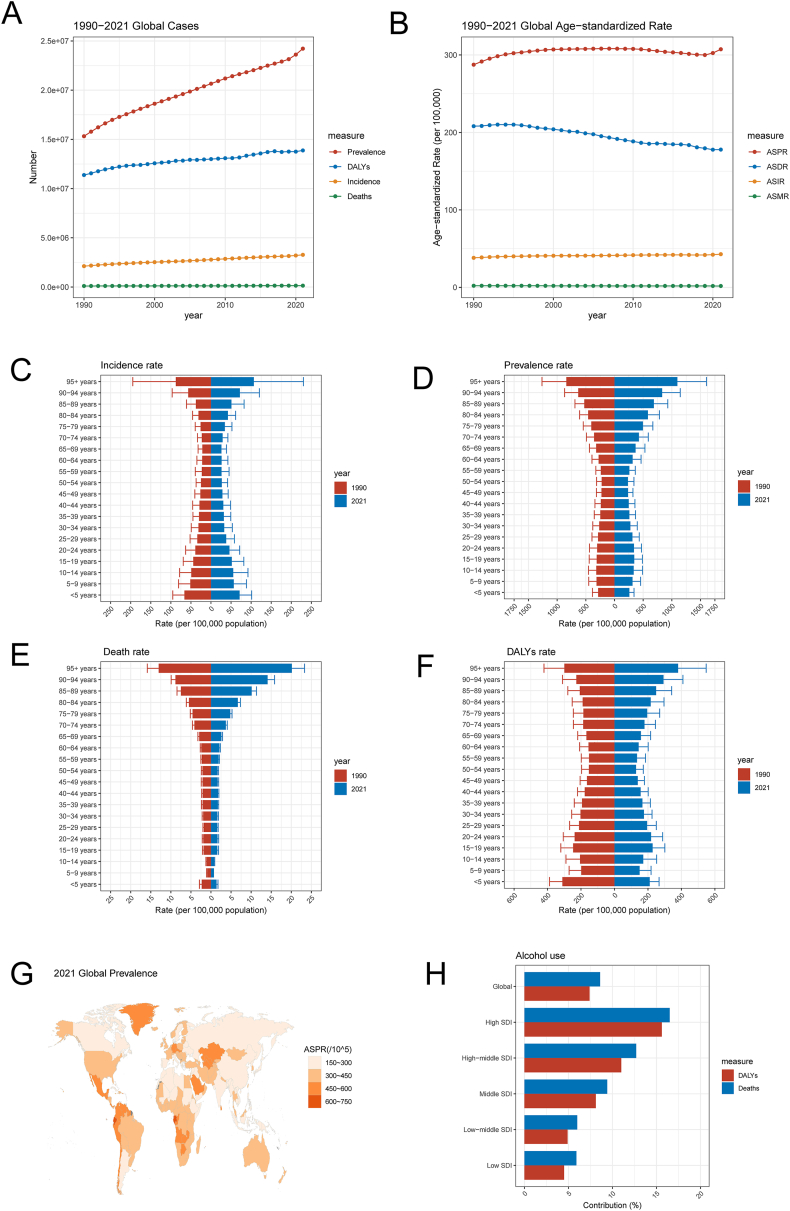
Alcohol consumption and epilepsy risk. **(A)** Global epilepsy cases in terms of prevalence, incidence, DALYs, and deaths from 1990 to 2021. **(B)** Global trends in the ASPR, ASDR, ASIR, and ASMR of patients with epilepsy from 1990 to 2021. **(C)** Crude incidence rates of epilepsy by age group in 1990 and 2021. **(D)** Crude prevalence rates of epilepsy by age group in 1990 and 2021. **(E)** Crude death rates of patients with epilepsy by age group in 1990 and 2021. **(F)** Crude DALY rates of patients with epilepsy by age group in 1990 and 2021. **(G)** Global map of epilepsy ASPR in 2021. **(H)** The percentage contribution of alcohol consumption to epilepsy DALYs and deaths in 2021, globally and by SDI quintiles. Abbreviations: ASIR, age-standardized incidence rate; ASPR, age-standardized prevalence rate; ASDR, age-standardized disability-adjusted life years rate; ASMR, age-standardized mortality rate; DALYs, disability-adjusted life years; SDI, sociodemographic index.

In this study, we conducted an initial global assessment of the potential risks of epilepsy using data from the 2021 GBD study. We analyzed the incidence, prevalence, number of deaths, and DALYs across regions, SDI quintiles, sex, and age groups. From 1990 to 2021, the incidence and prevalence of idiopathic epilepsy generally increased globally (Fig. 2A). The age-standardized incidence rate (ASIR) increased from 38.12 to 42.82 per 100,000, reflecting an average annual percentage change (AAPC) of 0.37% (Fig. 2B and Table 1). The number of prevalent cases increased from approximately 15.32 million in 1990 to 24.22 million in 2021, with the age-standardized prevalence rate (ASPR) increasing from 287.46 to 307.38 per 100,000 (Table S1). Notably, DALYs for idiopathic epilepsy increased from 11.38 million to 13.88 million, whereas the age-standardized DALY rate decreased from 208.08 to 177.84 per 100,000 (Table S2). Despite an increase in deaths from 0.10 million to 0.14 million for idiopathic epilepsy, the age-standardized mortality rate tended to decrease from 2.07 to 1.74 per 100,000 (Table S3). Globally, the burden of idiopathic epilepsy tended to increase for both males and females from 1990 to 2021. For females, the ASIR of idiopathic epilepsy increased from 36.12 per 100,000 in 1990 to 40.51 per 100,000 in 2021, corresponding to an AAPC of 0.36%. For males, the ASIR increased from 40.17 per 100,000 in 1990 to 45.11 per 100,000 in 2021, with a comparable AAPC of 0.37% (Table 1). When analyzed by age group, the highest burden of idiopathic epilepsy was observed in young children and older adults, with individuals aged 95 years and above exhibiting the highest crude incidence rates, a pattern consistent across crude prevalence, death, and DALYs (Fig. 2C–F).

**Table 1:** Age-standardized incidence rate and AAPC of idiopathic epilepsy at the global and regional levels, 1990–2021.

	Incidence (95% UI)				
	Cases in 1990 (million)	ASIR in 1990 (per 100,000)	Cases in 2021 (million)	ASIR in 2021 (per 100,000)	AAPC
Global	2121189 (1515389–2784,665)	38.12 (27.91–49.48)	3,272,734 (2,403,802–4125,119)	42.82 (31.24–53.72)	0.37 (0.34–0.39)
Gender					
Female	993,222 (711,206–1306,451)	36.12 (26.27–47.11)	1,536,119 (1,128,507–1948,582)	40.51 (29.41–50.98)	0.36 (0.34–0.39)
Male	1,127,967 (803,892–1473,788)	40.17 (29.5–51.85)	1,736,615 (1,279,500–2176,394)	45.11 (33.08–56.43)	0.37 (0.35–0.39)
SDI level					
High-middle SDI	350,385 (246,753–454,060)	33.4 (23.45–43.71)	426,210 (289,243–567,787)	37.05 (24.87–50.28)	0.33 (0.30–0.36)
High SDI	390,075 (268,615–511,963)	46.96 (32.25–62.14)	514,418 (342,283–684,799)	52.05 (34.37–70.31)	0.33 (0.25–0.41)
Low-middle SDI	464,657 (292,495–659,137)	36.09 (23.48–49.65)	798,512 (580,952–1032,876)	40.89 (30.19–52.23)	0.38 (0.35–0.41)
Low SDI	248,591 (140,511–368,640)	42.59 (24.16–63.41)	566,338 (370,984–776,083)	45.36 (30.11–60.72)	0.20 (0.17–0.22)
Middle SDI	665,330 (463,426–896,157)	36.37 (25.8–48.39)	964,574 (693,889–1233,906)	41.73 (29.83–53.3)	0.44 (0.41–0.47)
GBD region					
Central Europe, Eastern Europe, and Central Asia	160,022 (117,013–208945)	39.68 (28.73–51.71)	145,212 (100,228–193,226)	39.41 (27.06–52.75)	−0.04 (−0.09 to 0.01)
High-income	411,186 (278,236–540,413)	48.15 (32.98–64.12)	521,937 (347,791–693,196)	52.55 (34.76–71.65)	0.27 (0.19–0.35)
Latin America and Caribbean	279,151 (191,919–375,847)	65.93 (45.62–88.08)	366,402 (264,770–476,603)	63.22 (45.36–82.44)	−0.14 (−0.17 to −0.12)
North Africa and Middle East	167,812 (105,398–237,700)	42.98 (27.55–59.61)	298,247 (202,461–422,106)	47.37 (32.28–66.75)	0.30 (0.28–0.31)
South Asia	362,107 (223,755–517,303)	30.8 (19.05–43.33)	602,050 (428,444–784,538)	33.1 (23.43–42.49)	0.19 (0.05–0.33)
Southeast Asia, East Asia, and Oceania	439,777 (302,433–599,415)	25.46 (17.8–34.53)	625,602 (437,540–811,288)	31.46 (21.97–40.89)	0.69 (0.62–0.76)
Sub-Saharan Africa	301,134 (188,038–434,770)	52.86 (33.29–73.9)	713,285 (484,928–952,443)	56.85 (40.09–74.01)	0.24 (0.19–0.29)

Abbreviations: AAPC, average annual percentage change; ASIR, age-standardized incidence rate; SDI, sociodemographic index; UI, uncertainty interval.

When analyzed by country, Ecuador, Germany, and Equatorial Guinea had the highest ASPRs for idiopathic epilepsy. Specifically, Ecuador reported an ASPR of 94.94 per 100,000 people, Germany reported an ASPR of 91.82 per 100,000 people, and Equatorial Guinea reported an ASPR of 84.91 per 100,000 people (Fig. 2G). Detailed incidence, prevalence, mortality, and DALY data for each country can be found in Supplementary Table S4–S7. To explore the influence of alcohol use on the risk of idiopathic epilepsy, we evaluated the percentage contribution of alcohol use as a risk factor using 2021 GBD study data. The global contribution of alcohol use to the burden of idiopathic epilepsy, as measured by DALYs and deaths, is shown in Figure 2F. Alcohol use accounted for 7.41% of idiopathic epilepsy DALYs worldwide. The highest contribution was observed in the high SDI regions at 15.59%, whereas the lowest contribution was observed in the low SDI regions at 4.55% (Fig. 2H).

**Figure 3:**
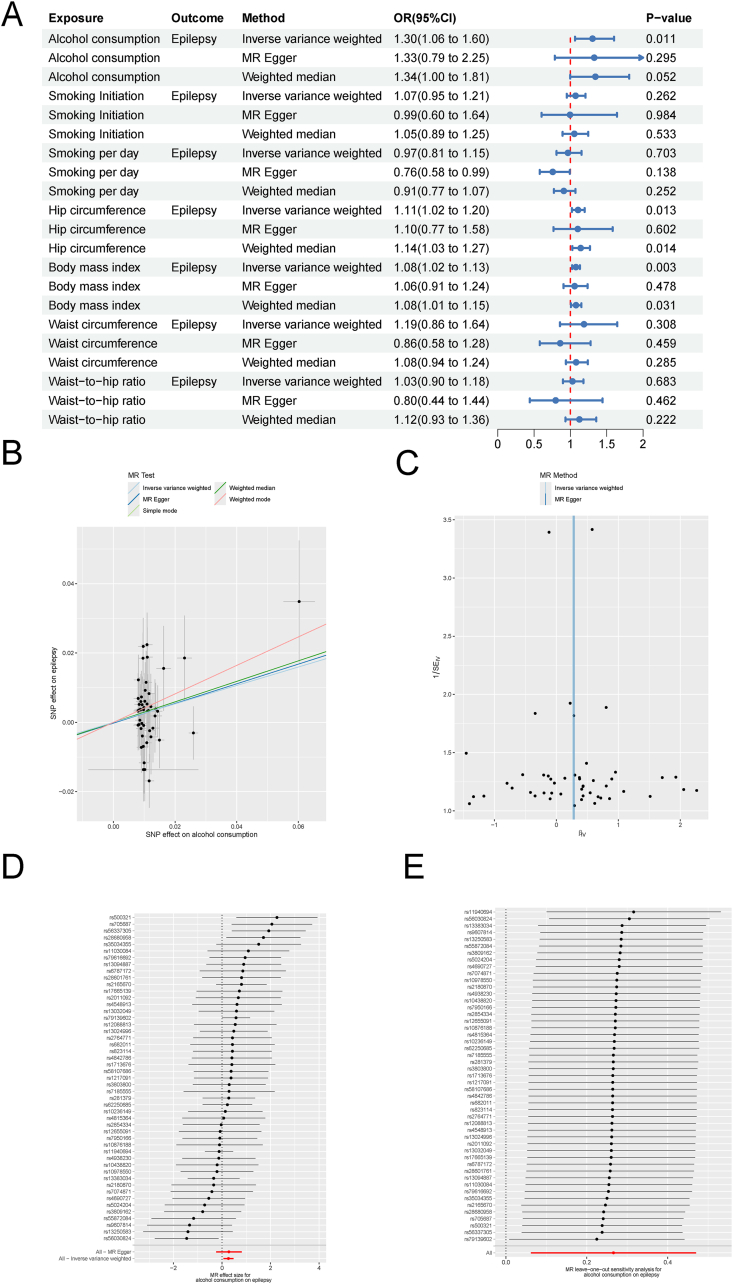
Causal link between alcohol consumption and epilepsy risk. **(A)** Summary of univariable MR analysis assessing causal relationships between alcohol consumption, smoking initiation, daily smoking status, hip circumference, body mass index, waist circumference, waist-to-hip ratio, and epilepsy. **(B)** Scatter plot from MR analysis of the effect of alcohol consumption on epilepsy. **(C)** Funnel plot from MR analysis of the effect of alcohol consumption on epilepsy. **(D)** Forest plot from MR analysis of the effect of alcohol consumption on epilepsy. **(E)** MR leave-one-out sensitivity analysis of the effect of alcohol consumption on epilepsy. Abbreviations: MR, Mendelian randomization.

Given the close relationship between alcohol consumption and epilepsy, verifying a true causal association for epilepsy prevention is essential. However, potential confounders such as smoking and obesity are also risk factors for epilepsy. To address these confounders, we performed a two-sample MR analysis to investigate the causal relationship between alcohol consumption and epilepsy. Details of the significant independent SNPs used in the MR analyses are provided in Supplementary Table S8. There was a significant causal relationship between alcohol consumption and epilepsy [odds ratio (OR) = 1.30, 95% confidence interval (CI) 1.06–1.60; *p* = 0.011; Fig. 3A–E]. Additionally, both hip circumference (OR = 1.11, 95% CI 1.02–1.20; *p* = 0.013) and BMI (OR = 1.08, 95% CI 1.02–1.13; *p* = 0.003) also exhibited significant causal associations with epilepsy (Fig. 3A). The MR-PRESSO test identified two outliers (rs62044525 and rs10506274) in the study of the effects of alcohol consumption on epilepsy, which were subsequently excluded from the analysis. Notably, the MR analysis results revealed no evidence of horizontal pleiotropy. However, the Q statistics indicated heterogeneity in the analysis of the effects of smoking initiation, hip circumference, and BMI on epilepsy (Table S9). Additionally, we aimed to further explore the relationship between alcohol consumption and epilepsy through multivariate MR analyses; however, an insufficient number of SNPs were identified. In summary, our integrated analysis of GBD 2021 risk factor data and MR results revealed that alcohol intake is associated with an increased risk of epilepsy, suggesting potential targets for therapeutic approaches.

### Molecular mechanisms underlying the relationship between alcohol consumption and epilepsy

**Figure 4:**
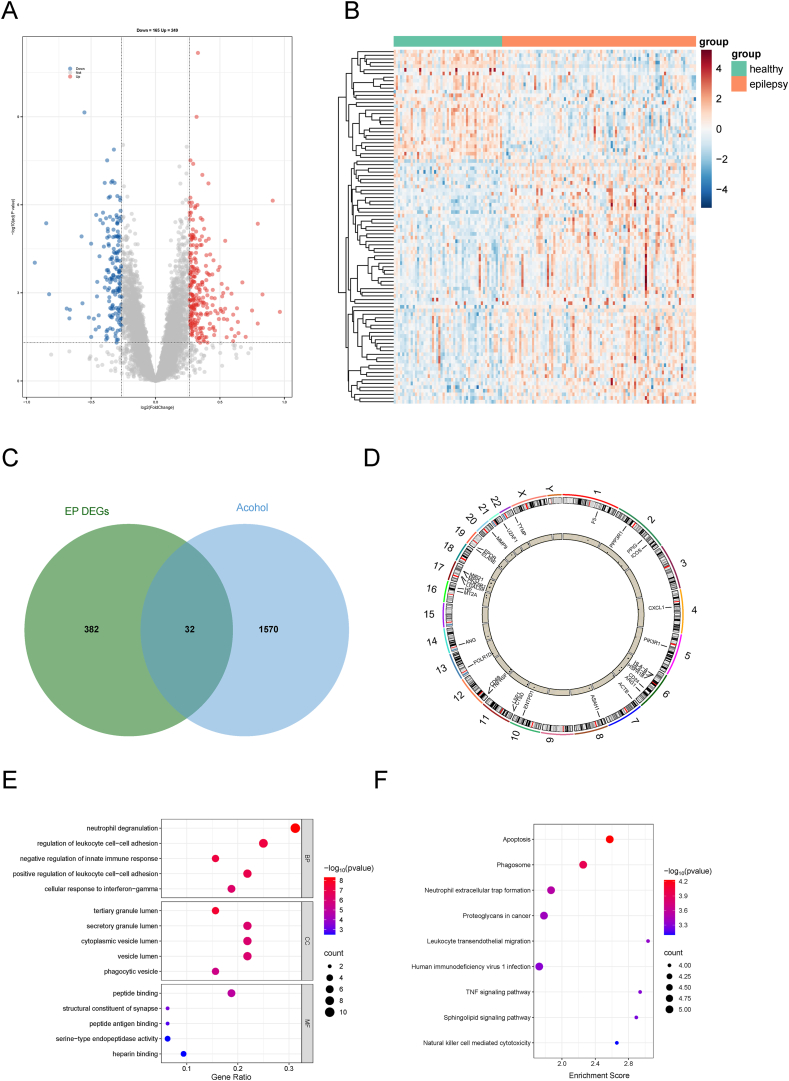
NET formation pathway in alcohol-induced epilepsy etiology. **(A)** Volcano plot of DEGs in epilepsy (GSE143272). **(B)** Heatmap of DEG expression in epilepsy. **(C)** Venn diagram of intersecting genes between epilepsy-related DEGs and alcohol-related genes. **(D)** Chromosomal locations of the 32 overlapping genes. **(E)** GO analysis of the overlapping genes. **(F)** KEGG analysis of the overlapping genes. Abbreviations: DEGs, differentially expressed genes; GO, Gene Ontology; KEGG, Kyoto Encyclopedia of Genes and Genomes; NET, neutrophil extracellular traps.

To elucidate the biological role of alcohol consumption in the etiology and progression of epilepsy, we conducted a DEG analysis using the GSE143272 dataset and integrated the identified DEGs with alcohol-related genes from GeneCards. We discovered 414 DEGs related to epilepsy. Specifically, 249 genes were up-regulated, whereas 165 were down-regulated (Fig. 4A and B). Additionally, we retrieved 1602 alcohol-related genes with relevance scores greater than 1 from GeneCards (Table S10). Venn diagram analysis revealed 32 overlapping genes (Fig. 4C), and their chromosomal locations are depicted in Figure 4D. We then conducted GO and KEGG enrichment analyses to explore the functions of these genes. GO analysis revealed the significant involvement of these 32 genes in neutrophil degranulation and the regulation of leukocyte cell–cell adhesion processes in the biological process category (Fig. 4E). In the cellular component category, the most enriched terms were tertiary granule lumen, secretory granule lumen, and cytoplasmic vesicle lumen (Fig. 4E). In the molecular function category, the predominant enriched terms were peptide binding and structural constituent of synapse (Fig. 4E). KEGG enrichment analyses revealed pathways such as apoptosis, phagosome, and NET formation (Fig. 4F).

**Figure 5:**
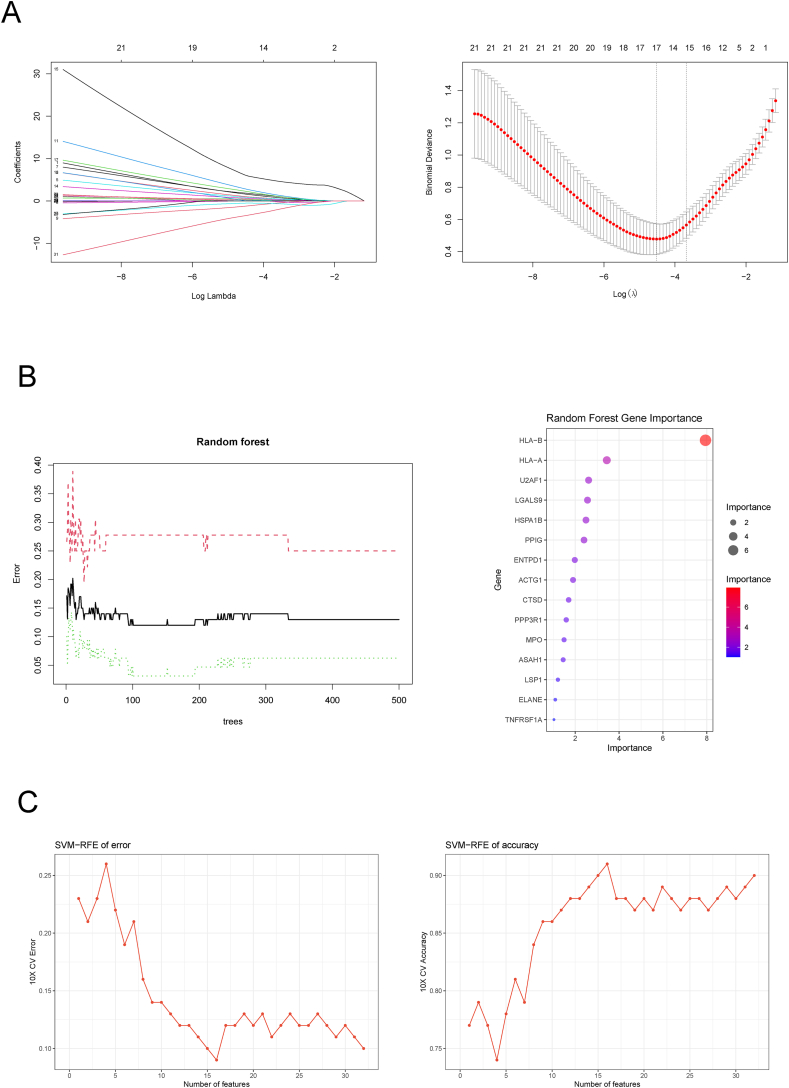
Identification of Hub Genes via Machine Learning. **(A)** LASSO regression for identifying key hub genes. **(B)** RF analysis for selecting hub genes. **(C)** SVM-RFE analysis for selecting hub genes. Abbreviations: LASSO, least absolute shrinkage and selection operator; RF, random forest; SVM-RFE, support vector machine-recursive feature elimination.

**Figure 6:**
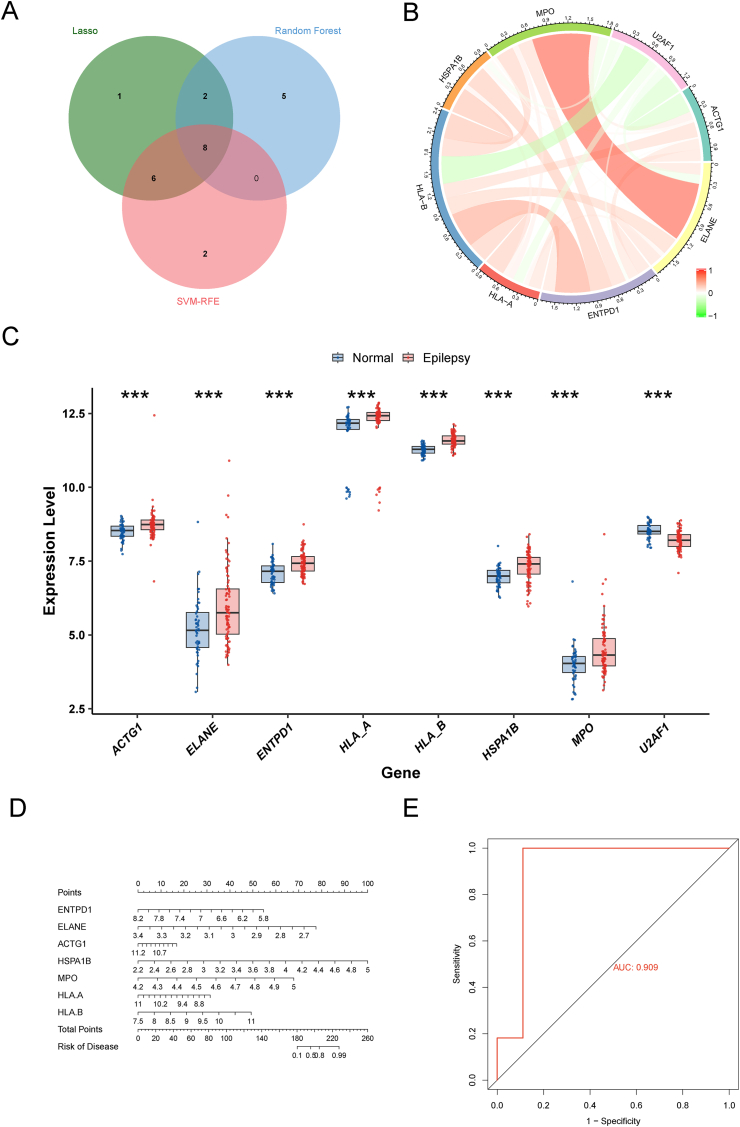
Validation of hub genes. **(A)** Venn diagram of intersecting genes from 3 machine learning algorithms in epilepsy. **(B)** Correlation analysis of 8 candidate biomarkers; green and red indicate negative and positive correlations, respectively. **(C)** Comparison of the expression of 8 candidate biomarkers between the normal and epilepsy groups in the GSE143272 dataset. ∗∗∗*p* < 0.001. **(D)** Nomogram based on the seven selected candidate biomarkers. **(E)** ROC curves for seven diagnostic markers in the epilepsy cohort (GSE16969 and GSE32534). Abbreviations: ROC, receiver operating characteristic.

To identify the key genes among the 32 overlapping genes derived from the Venn diagram, we applied ML analyses, including LASSO, RF, and SVM-RFE. LASSO regression revealed 17 genes among the 32 overlapping genes (Fig. 5A). The RF algorithm ranked the top 15 biomarkers on the basis of their importance (Fig. 5B). SVM-RFE analysis revealed 16 genes with the lowest error rate and highest accuracy after 10-fold cross-validation (Fig. 5C). Ultimately, the intersection of the results from these algorithms yielded the following candidate biomarkers: ACTG1, ELANE, ENTPD1, MPO, HLA-A, HLA-B, HSPA1B, and U2AF1 (Fig. 6A). The expression levels of these genes were strongly correlated in the GSE143272 dataset (Fig. 6B), with genes such as ACTG1, ELANE, ENTPD1, MPO, HSPA1B, HLA-A, and HLA-B exhibiting elevated expression levels, in contrast to the lower expression level observed for U2AF1 (Fig. 6C). Further validation was performed to confirm the reliability of these eight candidate diagnostic genes for epilepsy. However, U2AF1 was not identified in the validation set. Consequently, a nomogram was constructed based on the remaining seven genes, as shown in Figure 6D. Moreover, analysis of the receiver operating characteristic curves revealed that the seven genes used in the nomogram demonstrated good diagnostic performance in the epilepsy validation cohort, with an area under the curve of 0.909 (Fig. 6E). These findings confirm the potential of these seven candidate genes as key discriminatory markers for epilepsy diagnosis.

**Figure 7:**
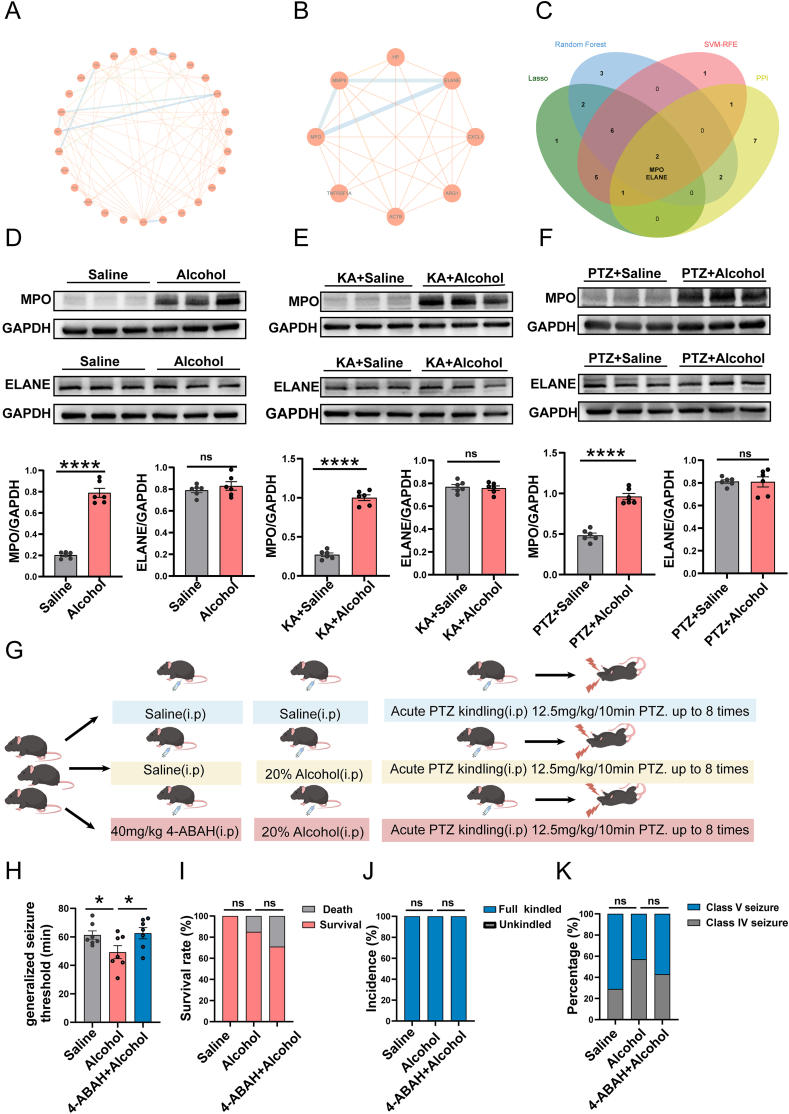
Role of MPO and ELANE in the risk of alcohol-induced epilepsy risk. **(A)** The PPI network illustrates the overall interactions of overlapping genes. **(B)** The PPI network presents the top 8 hub genes among the overlapping genes. **(C)** Venn diagram showing the overlapping genes identified using the LASSO, RF, SVM-RFE, and PPI methods. **(D)** Representative immunoblots and quantification of MPO and ELANE protein expression in mouse hippocampal lysates after acute alcohol exposure compared with those in the control group. *n* = 6; ∗∗∗∗*p* < 0.0001. **(E)** Representative immunoblots and quantification of MPO and ELANE protein expression in mouse hippocampal lysates after acute alcohol exposure combined with chronic KA-induced epilepsy. *n* = 6; ∗∗∗∗*p* < 0.0001. **(F)** Representative immunoblots and quantification of MPO and ELANE protein expression in mouse hippocampal lysates after acute alcohol exposure in the PTZ-induced seizure model group compared with those in the control group. *n* = 6; ∗∗∗∗*p* < 0.0001. **(G)** Schematic representation showing the experimental design. **(H)** Seizure latency in acute PTZ kindling model behavioral experiments. *n* = 7; ∗*p* < 0.05. **(I)** Survival rate in acute PTZ kindling model behavioral experiments. *n* = 7; ns, nonsignificant difference. **(J)** Kindling rate in acute PTZ kindling model behavioral experiments. *n* = 7; ns, nonsignificant difference. **(K)** Proportion of mice with a Racine score of IV-V in the acute PTZ-kindling model. *n* = 7; ns, nonsignificant difference. Statistical significance was determined by Student's *t*-test (D–F, H) or the χ^2^ test (I–K). Abbreviations: LASSO, least absolute shrinkage and selection operator; MPO, myeloperoxidase; RF, random forest; SVM-RFE, support vector machine-recursive feature elimination; PPI, protein–protein interaction.

Concurrently, a PPI network analysis was performed to elucidate the interactions among the 32 overlapping genes and to identify key node genes (Fig. 7A and B). Finally, the convergence of insights from ML analyses and PPI network analysis revealed the candidate biomarkers, which were visualized in a Venn diagram (Fig. 7C), highlighting MPO and ELANE.

### Role of MPO and ELANE in alcohol-induced epilepsy risk

To investigate the potential roles of MPO and ELANE in the risk of alcohol-related epilepsy, we first assessed how acute alcohol exposure affects their protein expression levels in the brain. The results revealed that acute alcohol exposure significantly increased MPO expression but did not affect ELANE protein levels (Fig. 7D). We further explored the impact of acute alcohol use on epilepsy using two models: chronic KA-induced and acute PTZ-induced epilepsy. Western blot analysis revealed that, in both models, acute alcohol exposure up-regulated MPO protein expression, whereas ELANE protein levels remained unchanged (Fig. 7E and F).

To further elucidate whether MPO dynamics are correlated or play a functional role in alcohol-induced epilepsy, we examined the effect of modulating MPO activity on seizure susceptibility using a PTZ kindling model. Initial experiments demonstrated that compared with saline treatment, alcohol treatment significantly shortened seizure latency, although no significant differences were observed in the induction rate, survival rate, or proportion of mice with a Racine score of IV-V. Notably, compared with alcohol-treated mice, pretreatment with the MPO inhibitor 4-ABAH prior to alcohol exposure significantly prolonged seizure latency, whereas comparable outcomes in terms of the induction rate, survival rate, and seizure severity were maintained. These findings collectively suggest that MPO positively regulates alcohol-induced epileptic seizures (Fig. 7G–K, partly created using BioGDP.com).

## Discussion

Using integrated multiomics approaches coupled with preliminary experimental validation, we revealed novel insights into the complex interplay between alcohol consumption and epilepsy, particularly through the MPO-mediated NETosis pathway. Furthermore, we also revealed that 4-ABAH is a candidate drug for mitigating alcohol-exacerbated epilepsy.

Conflicting conclusions about whether alcohol consumption plays a positive or negative role in seizure regulation have raised concerns. Most previous studies have shown that alcohol consumption has a negative effect on epilepsy. Two meta-analyses of case‒control studies demonstrated that individuals who drank alcohol had an increased risk of epilepsy compared with those who did not, and that there was a significant positive dose‒response relationship between the amount of alcohol consumed and the risk of developing epilepsy.^13^^,^^14^ However, some cohort studies have reached inconsistent conclusions. The Nurses' Health Study II revealed that moderate alcohol intake was not associated with epilepsy.^10^ A multicenter study revealed that moderate alcohol intake was associated with a lower risk of late-onset epilepsy.^17^ These inconsistencies may be attributed to differences in study populations and designs. Additionally, the results of these studies are inevitably influenced by confounding factors. In this study, we integrated data from the 2021 GBD study with MR analysis, complemented by behavioral experiments in a mouse epilepsy model. We found that alcohol consumption is a risk factor for epilepsy and that a significant causal relationship exists between the two. Additionally, acute alcohol exposure increased the susceptibility to epileptic seizures in epileptic mice. These findings are supported by previous epidemiological studies and experimental evidence. A GBD study conducted in Latin America and the Caribbean between 1990 and 2019 identified alcohol use as one of the important determinants of epilepsy-related burden.^48^ Furthermore, a study investigating key epileptogenic mechanisms in alcohol-related seizure development demonstrated that chronic ethanol treatment significantly increased seizure frequency over time in C57BL/6 mice.^49^

Using integrated bioinformatics analysis, this study further revealed NET formation as a core pathological mechanism linking alcohol consumption to increased epilepsy risk. Previous research has shown that NET formation is tightly regulated. Under physiological conditions, NETs perform functions such as antibacterial defense, immune cell recruitment, and inflammation containment, thus playing crucial roles in maintaining homeostasis.^20^^,^^50^ However, excessive alcohol consumption triggers a cascade of aberrant NET responses through multiple interconnected pathways. First, alcohol disrupts intestinal barrier integrity, permitting the translocation of endotoxins such as lipopolysaccharides into the bloodstream, where they synergize with reactive oxygen species (ROS) to induce NETosis.^51^ Second, chronic ethanol intake is primarily metabolized via cytochrome P450 2E1.^52^ Its overexpression accelerates ethanol oxidation, resulting in the excessive production of acetaldehyde and ROS, which may further promote NET release. These alcohol metabolites and ROS activate neutrophils, triggering cellular activation and granule release. Under physiological conditions, activated neutrophils release NETs to capture pathogens. However, excessive alcohol consumption disrupts intracellular redox homeostasis, leading to the overproduction of ROS that directly damage neutrophil DNA and membrane structures and precipitate excessive NET formation. Uncontrolled NET release not only fails to effectively clear pathogens but also leads to the accumulation of inflammatory mediators, exacerbating inflammatory responses and tissue injury.^53^^,^^54^ Importantly, the infiltration of abnormal peripheral NETs into the brain may increase the likelihood of seizures.^55^^,^^56^ Abnormal NETs are laden with cytotoxic proteins that can damage neuronal synapses and disrupt neurotransmitter transmission. Additionally, the DNA components within abnormal NETs activate immune cells in the brain, causing the continuous release of inflammatory mediators.^57^^,^^58^ This continuous release of inflammatory mediators induces excessive neuronal excitation and abnormal electrical discharge, forming the pathological basis for seizures. Finally, abnormal NETs also interfere with the function of cerebral vascular endothelial cells,^59^ affecting cerebral blood perfusion and thereby further facilitating the onset and progression of epilepsy.

Using ML techniques, we identified two genes that are up-regulated in the investigation of the mechanisms underlying alcohol-exacerbated epilepsy: MPO and ELANE. Further validation using molecular experiments combined with animal behavioral testing ultimately confirmed the critical role of MPO. Previous research has demonstrated that MPO plays crucial roles in NET formation and is involved in maintaining the normal physiological functions of neutrophils.^60^ NET formation primarily occurs through a form of cell death known as NETosis.^61^ During NETosis, the nuclear membrane of neutrophils disintegrates, and chromatin is depolymerized within the cytoplasm, and combines with cytoplasmic and granular components. The cell membrane gradually becomes permeable, enabling NETs to be released from the cell. The MPO protein is actively involved in this process.^20^^,^^62^ Specifically, when the MEK-extracellular signal-regulated kinase signaling pathway induces ROS production, the MPO pathway becomes activated, promoting chromatin decondensation and suppressing phagocytosis. Consequently, MPO may serve as a therapeutic target for alcohol-related epilepsy.

Previous studies have progressively revealed the pathogenic role of MPO and its inhibitor 4-ABAH in epilepsy. MPO-mediated oxidative stress has been established as a pivotal mechanism driving pathological dysfunction in the epileptic brain.^63^ Experimental evidence revealed significant elevation of cerebral MPO levels during pilocarpine-induced epileptogenesis in murine models.^47^ Importantly, pharmacological inhibition of MPO activity delays spontaneous recurrent seizure onset, mitigates seizure severity, and inhibits aberrant mossy fiber sprouting.^47^ These findings were further corroborated by studies demonstrating that biochanin A pretreatment significantly attenuated chronic PTZ-induced epileptogenesis while concomitantly reducing MPO protein expression.^64^ Collectively, these findings demonstrate that MPO exacerbates epileptic neuropathology through the dual mechanisms of neutrophil-mediated neuroinflammation and oxidative stress pathways, positioning MPO as a critical mediator of epilepsy-associated brain damage.^65^ Our findings are consistent with those of previous studies, demonstrating that acute alcohol exposure significantly increases MPO expression. Notably, elevated MPO protein levels were also observed during both the chronic phase of KA-induced epilepsy and the acute phase of PTZ-induced epilepsy following acute alcohol exposure. In behavioral experiments using an acute PTZ-induced epilepsy model, we found that the MPO inhibitor 4-ABAH significantly prolonged seizure onset latency under conditions of acute alcohol exposure.

This study elucidates the pathogenesis of alcohol-related epilepsy through comprehensive multiomics analysis. We have identified the key gene MPO as a potential biomarker and MPO inhibitors as promising therapeutic candidates for alcohol-induced epilepsy. However, several notable limitations should be acknowledged. First, while the MR analysis was primarily conducted in participants of European descent, the bioinformatics analysis included both Asian and European populations. This discrepancy may introduce confounding factors due to ethnic variations, potentially compromising the accuracy of the results. Second, this study focused exclusively on the role of MPO in mediating the effects of acute alcohol exposure on PTZ-induced acute seizures and did not investigate its involvement in chronic alcohol exposure across various epilepsy models. Nevertheless, our current findings undeniably support the critical role of MPO in alcohol-triggered epileptogenesis. Finally, although our study highlights the role of MPO in the NET pathway, importantly, this represents only a subset of the broader NET formation mechanisms. Future research should explore additional components of the NET pathway to provide a more comprehensive understanding of its involvement in the relationship between alcohol consumption and epilepsy risk.

## Conclusion

In summary, our integrated multi-omics study combined with experimental validation demonstrates that alcohol consumption indeed exacerbates epileptic seizures, with the MPO-mediated NETosis pathway playing a pivotal role in the alcohol–epilepsy relationship. These findings not only advance our understanding of the association between alcohol use and epilepsy, but also facilitate the development of novel therapeutic strategies for alcohol-related epilepsy. Further research is warranted to validate these findings in clinical settings and explore the therapeutic potential of the identified genetic targets.

## CRediT authorship contribution statement

**Ningning Zhang:** Writing – review & editing, Writing – original draft, Visualization, Validation, Methodology, Formal analysis, Data curation, Conceptualization. **Sirui Chen:** Writing – review & editing, Writing – original draft, Methodology, Formal analysis, Data curation, Conceptualization. **Jialing Jiang:** Conceptualization, Data curation, Formal analysis, Methodology, Validation, Visualization, Writing – original draft, Writing – review & editing. **Hong Jiang:** Conceptualization, Data curation, Formal analysis, Methodology, Validation, Visualization, Writing – original draft, Writing – review & editing. **Qing Wang:** Writing – review & editing, Writing – original draft, Formal analysis, Data curation, Conceptualization. **Srikrishnan Raju:** Writing – review & editing, Writing – original draft, Formal analysis, Data curation, Conceptualization. **Jackson G. Schumacher:** Writing – review & editing, Writing – original draft, Formal analysis, Data curation, Conceptualization. **Jiliang Lu:** Resources, Methodology, Formal analysis. **Yihe Lian:** Resources, Methodology, Formal analysis. **Yuansong Zhang:** Resources, Methodology, Formal analysis. **Yuanhang Xu:** Resources, Methodology, Formal analysis. **Lan Zhang:** Resources, Investigation, Formal analysis. **Yaqing Liu:** Resources, Investigation, Formal analysis. **Junqiang Li:** Resources, Investigation, Formal analysis. **Yiru Zhang:** Writing – review & editing, Methodology. **Yuxuan Wang:** Writing – review & editing, Methodology. **Yixue Gu:** Conceptualization, Project administration, Resources, Validation, Writing – review & editing. **Tiancheng Wang:** Writing – review & editing, Validation, Supervision, Resources, Project administration, Methodology, Funding acquisition, Formal analysis. **Xin Tian:** Writing – review & editing, Validation, Supervision, Resources, Project administration, Formal analysis, Funding acquisition, Methodology.

## Ethics declaration

The use and care of experimental animals were approved by the Research Ethics and Regulations Committee of Chongqing Medical University, Chongqing, China. All experimental procedures followed the approved guidelines.

## Data availability statement

Part of the analyses were conducted using publicly available data.

## Funding

This work was supported by grants from the National Natural Science Foundation of China (No. 82160262, 82571663, 82001378), General Project of Gansu Province Joint Research Fund (China) (No. 23JRRA1504), Major Scientific Research Projects of Scientific and Technological Innovation in Health Industry of Gansu Province in 2025 (China) (No. GSWSZD2025-12), Natural Science Foundation of Chongqing (China) (No. CSTB2023NSCQ-JQX0035, CSTB2022NSCQ-LZX0038), and Chongqing Postdoctoral Research Project Special Funding (No. 2023CQBSHTBT001).

## Conflict of interests

Xin Tian is an editorial board member for *Genes & Diseases* and was not involved in the editorial review or the decision to publish this article. All authors declare that there are no other competing interests.
